# Novel regulation pathway of eclosion hormones in *Tribolium castaneum* by distinct transcription factors through the initiation of 20-hydroxyecdysone

**DOI:** 10.1016/j.jbc.2024.107898

**Published:** 2024-10-17

**Authors:** Huiling Zhou, Gaoke Lei, Yusi Li, Peng Chen, Zhiping Liu, Chengjun Li, Bin Li

**Affiliations:** 1 College of Life Sciences, Nanjing Normal University, Nanjing, China; 2 Institute of Plant Protection, Fujian Academy of Agricultural Sciences, Fuzhou, China

**Keywords:** *Tribolium castaneum*, eclosion hormone, transcriptional regulation, ecdysone, growth and development

## Abstract

Eclosion hormone (EH) is not only a key trigger of insect ecdysis, but is also involved in the regulation of important physiological processes such as development, diapause, metamorphosis, and reproduction. EH is an ideal target for RNAi treatment and prevention of the *Tribolium castaneum*. However, two *EH* genes in *T. castaneum* demonstrate distinct replication and functional conversion relationships, and the mechanisms of transcriptional regulation of *EH* remain largely unexplored and poorly understood. In this study, the activity of highly active promoter fragments and potential transcription factors of *TcEH* and *TcEHL* were identified using the Dual-Luciferase reporter system and TANSFAC. *TcSlbo* and *TcCAD* were revealed to be important transcription factors for *TcEH* and *TcEHL*, respectively. Knockdown of *TcSlbo* failed to slough off the old epidermis of *T. castaneum* and prevented them from developing into adults. Furthermore, we demonstrated for the first time that 20-hydroxyecdysone affects the expression of *TcEH* and *TcEHL* by regulating the transcriptional activities of *TcSlbo* and *TcCAD*. This study provides new insights into the transcription regulation of *TcEH* and *TcEHL*, their roles in insect growth and development, and the involvement of 20-hydroxyecdysone in eclosion regulation, offering potential molecular targets for future pest management strategies.

*Tribolium castaneum* (Coleoptera: Tenebrionidage) poses a significant threat to the global food supply chain, particularly in food storage and food safety (1, 2). As a major pest of stored grains, including wheat, maize, and rice, *T. castaneum* causes direct losses by consuming and contaminating these grains and further exacerbates the problem with its rapid reproductive capacity (3). *T. castaneum* has also developed significant resistance to a number of commonly used insecticides, such as organophosphates and pyrethroid (4, 5). This resistance not only makes management more difficult and costly for agricultural producers but it can also indirectly threaten human health through contamination. Therefore, there is an urgent need to develop new, safer, and more effective pest control technologies, particularly strategies based on RNAi technology. RNAi technology offers great potential for pest control due to its precision, environmental friendliness, and biosafety (6). RNAi with specific target genes effectively affects the growth and development of the *T. castaneum*, showing good prospects for its prevention and control (7). However, it has been shown that pest resistance to dsRNA can reduce the effectiveness of RNAi-based control, highlighting the need to identify new molecular targets for pest management (8).

Eclosion hormone (EH) is a peptide hormone produced in brain neurosecretory cells and stored in the lateral body of heart and abdominal ganglia (9). EH serves as a key trigger for insect eclosion and is also involved in the regulation of important physiological processes such as development, diapause, metamorphosis, and reproduction (10). This neuropeptide hormone has been identified in a wide range of insects and other arthropods, with the number of *EH* genes varying among species due to gene duplication and evolution. For instance, three *EH* genes have been identified in both *Aedes egypti* (11) and *Acyrthosiphon pisum* (12). Two homologous genes of *EH*, *TcEH*, and *TcEHL* (*EH*-like), are present in the *T*. *castaneum*. Interestingly, *TcEH*, which was previously thought to play a central role in *T. castaneum* eclosion, may represent an ancient and conserved gene copy (13, 14). However, recent phylogenetic and gene structure analyses have shown that *TcEH* is derived from the replication of *TcEHL*. Following this duplication, *TcEH* underwent functional differentiation from *TcEHL*. *TcEH* appears to have reverted to an ancestral functional, playing a central role in eclosion regulation, while *TcEHL* has acquired a new functional (15, 16). Despite these findings, the transcriptional regulation of *TcEH* and *TcEHL* remains largely unexplored.

Neuropeptide hormones precisely regulate insect eclosion (17, 18). Transcriptional regulation serves as a critical link between gene expression and physiological regulation (19). Recent studies have revealed that transcription factors (TFs) are involved in larval molting and eclosion through a regulatory network of the eclosion. Knockdown of the TF *βftz-f1* prevents the release of ecdysis triggering hormone (ETH) from Inka cells, leading to developmental arrest and multiple defects in *Drosophila*. However, these larvae can be rescued by precisely timed ETH injections or targeted expression of *βftz-f1* (20). Similarly, knocking down *SoxC* gene expression caused eclosion defects in *T. castaneum* and *Spodoptera frugiperda*. While, only the SfSoxC protein has been shown to bind directly to the *SfEH* promoter (21). The transcriptional regulatory mechanism of *TcEH* and *TcEHL* remains poorly understood. Is *SoxC* a direct regulator factor of *TcEH* and *TcEHL*? Are there other TFs that regulate the expression of *TcEH* and *TcEHL*. How these TFs affect eclosion remains to be investigated.

The classical insect molting hormone 20-hydroxyecdysone (20E) also plays a critical role in insect molting (22, 23). Injection of 20E into *Exopalaemon carinicauda* increased *EcEH* expression and promoted eclosion (24). The *LdEH* expression of *Leptinotarsa decemlineata* was also positively correlated with 20E titers, and 20E rescued the disruption of EH-induced molt inhibition effects (25). In addition, cGMP, a second messenger mediating EH signaling, has been identified as a participant in ecdysone synthesis (26, 27). However, whether there is a direct or indirect regulatory relationship between 20E and EH and how 20E regulates EH to participate in insect eclosion remains to be investigated.

In this study, we identified *TcSlbo* and *TcCAD* as key response factors to 20E for the first time. These TFs directly bind to the promoter regions of the *TcEH* and *TcEHL* genes, respectively, acting as direct regulators. Notably, knockdown of *TcSlbo* resulted in pupae failing to successfully shed their old epidermis, preventing their development into adults. This study not only elucidates the TFs of the *TcEH* and *TcEHL* genes and reveals their key roles in regulating insect development and physiological functions but also provides potential molecular targets for future pest management strategies.

## Results

### Screening for *TcEH/TcEHL* TFs

**Figure 1:**
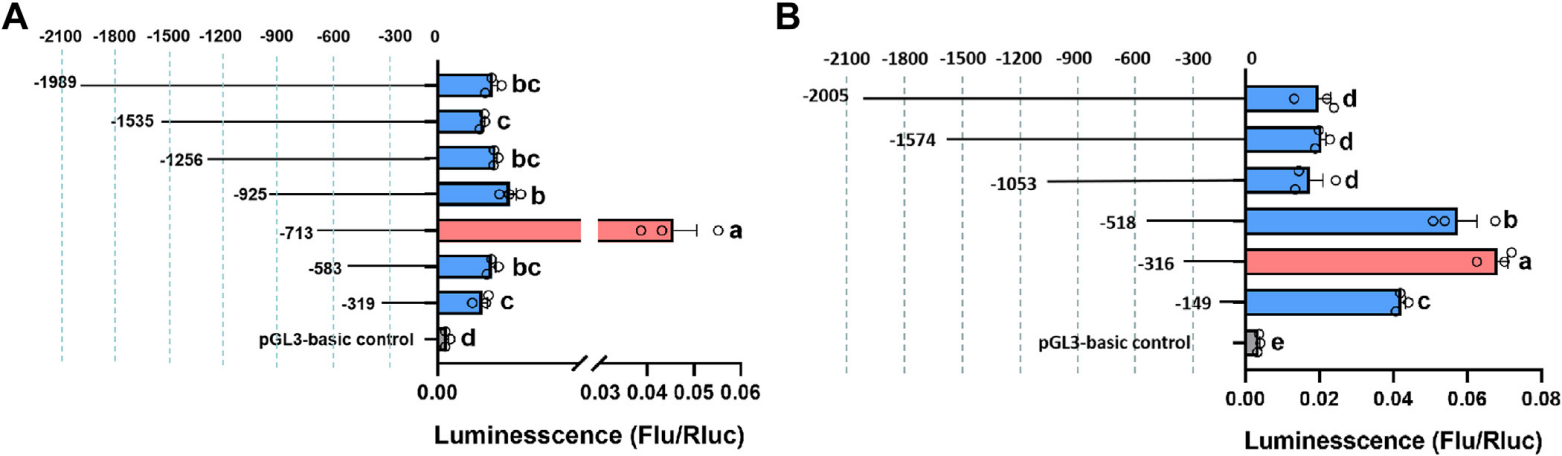
**Screening for highly active fragments of the *TcEH/TcEHL* promoter.***A*, transcriptional activity of the *TcEH* progressive deletion promoter was detected using a Dual-Luciferase reporter system. *B*, transcriptional activity of the *TcEHL* progressive deletion promoter was detected using the Dual-Luciferase reporter system. The pGL3-basic plasmid was used as a control. Data were presented as the mean ± standard errror (one-way ANOVA, Tukey's, *p* < 0.05, n = 3).

TFs regulate the expression of target genes by binding to different segments of their promoter. In this study, we amplified sequences approximately 2 kb upstream of the transcription start site of the *TcEH* and *TcEHL* genes using the *T. castaneum* genomic database (https://ibeetle-base.uni-goettingen.de) and evaluated the activity of the different promoter segments using the Dual-Luciferase reporter assay system. The results showed that the promoter fragment (−713/+1 bp) of *TcEH* had the highest activity, while the segments from −316 to +1 bp of the *TcEHL* promoter demonstrated the highest activity. Further analysis revealed that the core promoter region of the *TcEH* is located at −583 bp to −713 bp, and the core promoter region of the *TcEHL* is located at −149 bp to −316 bp (Fig. 1, *A* and *B*).

**Figure 2:**
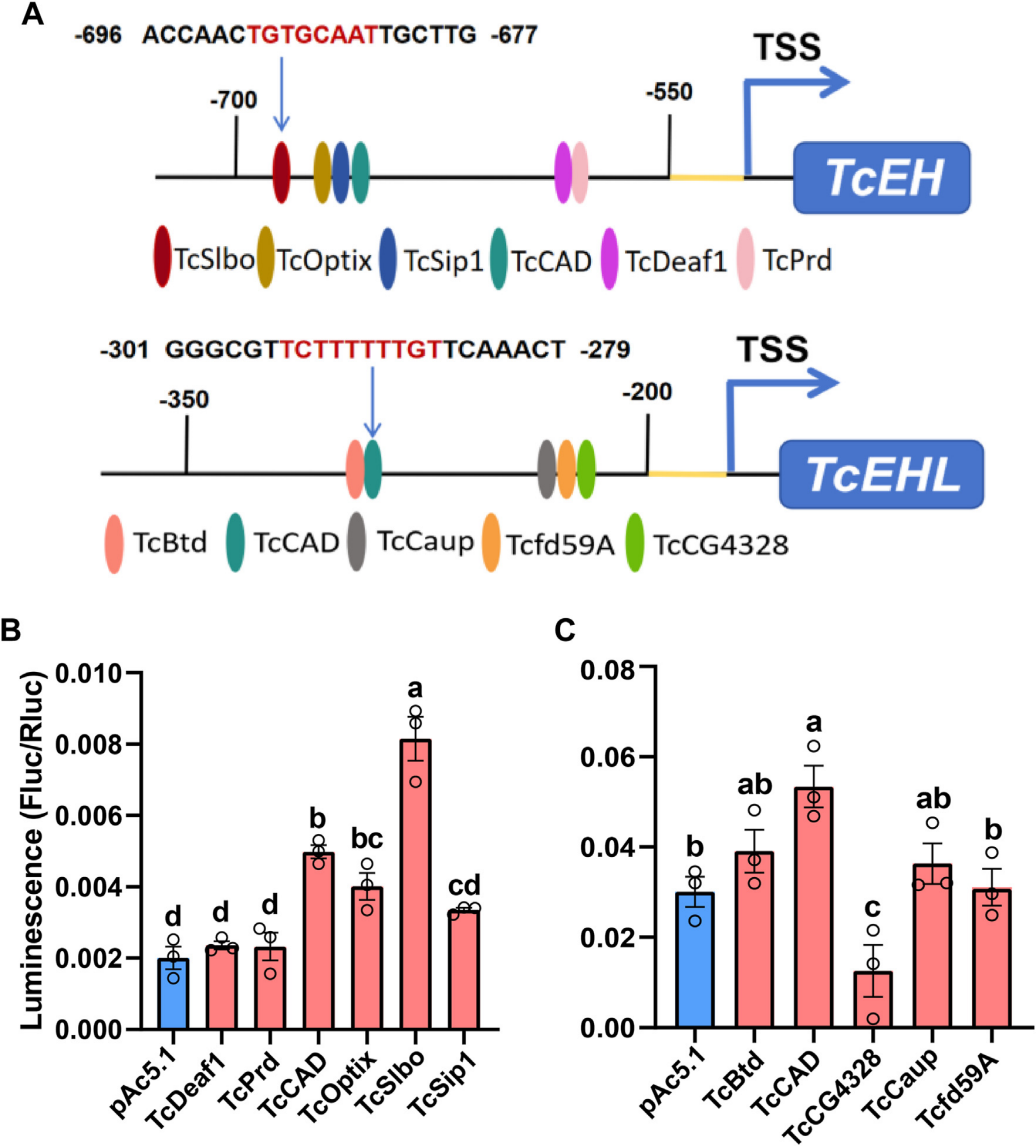
**Screening of the *TcEH*/*TcEHL* transcription factors.***A*, schematic representation of potential transcription factor binding sites in the *TcEH*/*TcEHL* promoter region. CREs were represented by different coloured ovals. *B*, effects of potential transcription factors on *TcEH* promoter activity were detected using the Dual-Luciferase reporter assay system. *C*, effects of potential transcription factors on *TcEHL* promoter activity were detected using the Dual-Luciferase reporter assay system. The pAc5.1 plasmid was used as a control. Data were presented as the mean ± SE (one-way ANOVA, Tukey's, *p* < 0.05，n = 3). CRE, *cis*-regulatory element; TSS, transcription start site.

To identify TFs that may interact with these core promoter regions, we used the TRANSFAC database (http://gene-regulation.com/) for predictive analysis. The results suggested that six TFs could bind to the *TcEH* promoter, and five TFs could bind to the *TcEHL* promoter (Fig. 2*A*; Table S3). To validate these predictions, we cloned the coding sequences of the predicted TFs into the pAc5.1 b plasmid and performed overexpression experiments in S2 cells. The effects of the TFs on the transcriptional activity of the *TcEH* and *TcEHL* promoters were assessed using the Dual-Luciferase reporter assay. The results showed that the activity of the *TcEH* promoter was significantly increased in S2 cells by overexpressing *TcCAD*, *TcOptix*, and *TcSlbo*. At the same time, the activity of the *TcEHL* promoter was significantly increased in S2 cells by overexpressing *TcBtd*, *TcCAD*, and *TcCaup* (Fig. 2, *B* and *C*).

### Effects of potential *TcEH/TcEHL* TFs on eclosion rate and adult lifespan of the *T. castaneum*

**Figure 3:**
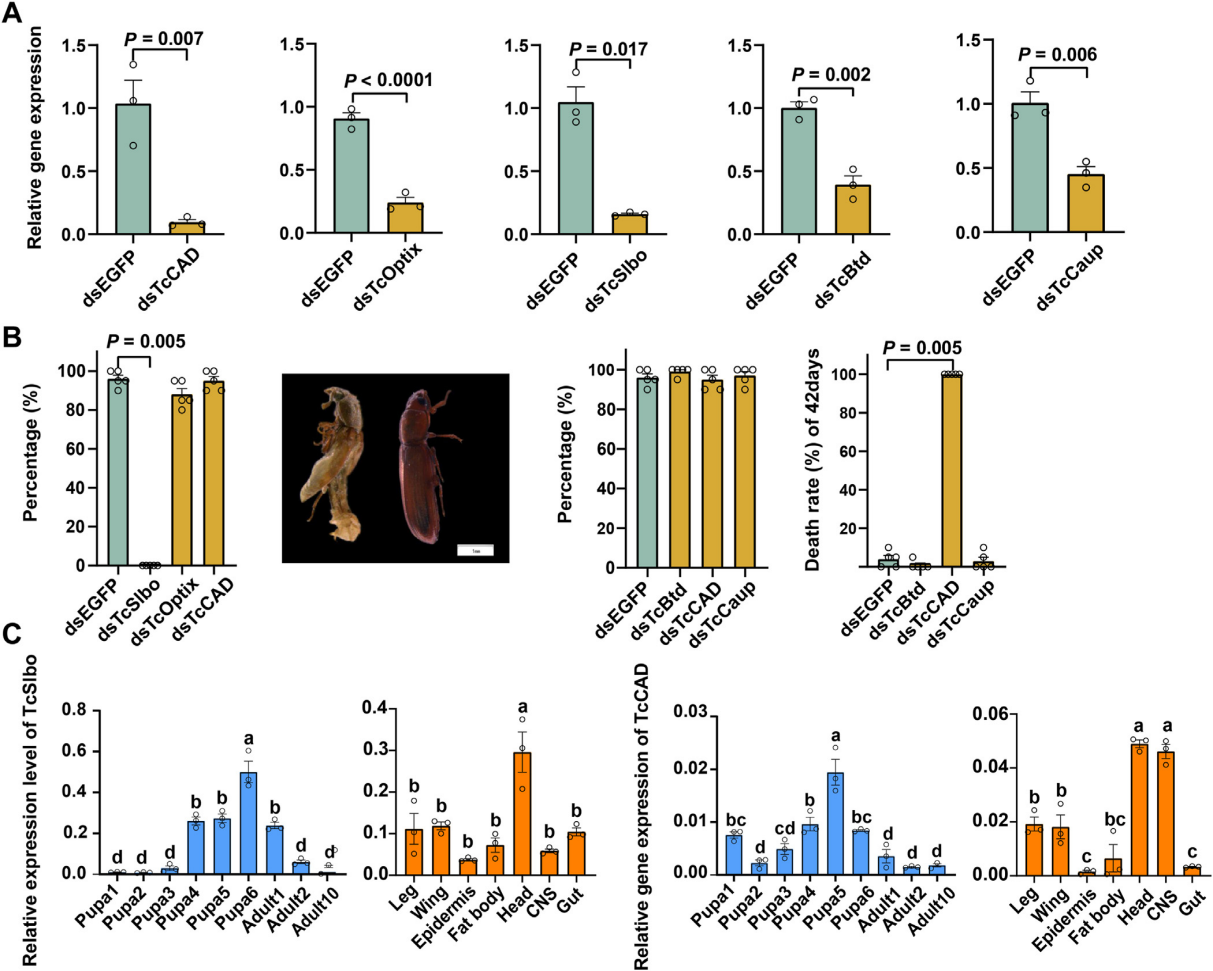
***TcSlbo* and *TcCAD* were the transcription factors of *TcEH* and *TcEHL*, respectively.***A*, silencing efficiency of potential *TcEH*/*TcEHL* transcription factors. Data were presented as the mean ± SE (independent samples T, *p* < 0.05, n = 3). *B*, effects of potential *TcEH/TcEHL* transcription factors on eclosion rate and adult lifespan of the red flour beetle. Data were presented as the mean ± SE (independent samples T, *p* < 0.05, n = 5). *C*, spatio-temporal expression patterns of *TcSlbo* and *TcCAD* transcription factors (one-way ANOVA, Tukey's HSD, *p* < 0.05, n = 3).

To further investigate the roles of TFs of *TcEH* and *TcEHL*, we used RNAi technology to reduce the expression of these TFs, including *TcSlbo*, *TcCAD*, *TcOptix*, *TcBtd*, and *TcCaup*. The efficiency of silencing the different TFs was evaluated 3 days after dsRNA injection in *T*. *castaneum*, yielding knockdown efficiencies of 84.85% for *TcSlbo*, 90.77% for *TcCAD*, 73.48% for *TcOptix*, 61.63% for *TcBtd*, and 54.74% for the *TcCaup* (Fig. 3*A*). Further monitoring of the eclosion rate and adult lifespan revealed that 6-day-old pupae with silenced *TcSlbo* showed 100% failure to eclose. Knockdown of *TcSlbo* resulted in head eversion, deformed wings and incomplete molting, and ultimately resulted in death (Fig. 3*B*). However, the eclosion rate of knockdown *dsTcOptix* and double-stranded *TcCAD* (*dsTcCAD)* was not significantly different from that of the control group. For the *TcEHL* TFs, downregulation of their expression did not significantly affect the eclosion rate. However, all *TcCAD*-knockdown red flour beetles died within 42 days and had a significantly shortened lifespan. The phenotypes observed with knockdown of *TcSlbo* and *TcCAD* were consistent with those observed with knockdown of *TcEH* and *TcEHL* (15, 28). These results suggest that *TcSlbo* and *TcCAD* can activate the transcription of *TcEH* and *TcEHL* by binding to their promoters, thereby playing a crucial role in the physiological development of the red flour beetle.

### Spatio-temporal expression patterns of the TFs *TcSlbo* and *TcCAD*

To further understand the roles of the *TcSlbo* and *TcCAD* TFs, we analyzed their expression patterns in different developmental stages and tissues. The results showed that the expression of *TcSlbo* and *TcCAD* increased significantly in the late pupal stage. In addition, both TFs were highly expressed in the central nervous system (CNS) and head (Fig. 3*C*). The spatio-temporal expression patterns of the TFs *TcSlbo* and *TcCAD* were similar to those of *TcEH* and *TcEHL*, providing further support for the hypothesis that *TcSlbo* and *TcCAD* serve as TFs for *TcEH* and *TcEHL*, respectively.

### *TcSlbo* and *TcCAD* in regulating cGMP levels during eclosion

**Figure 4:**
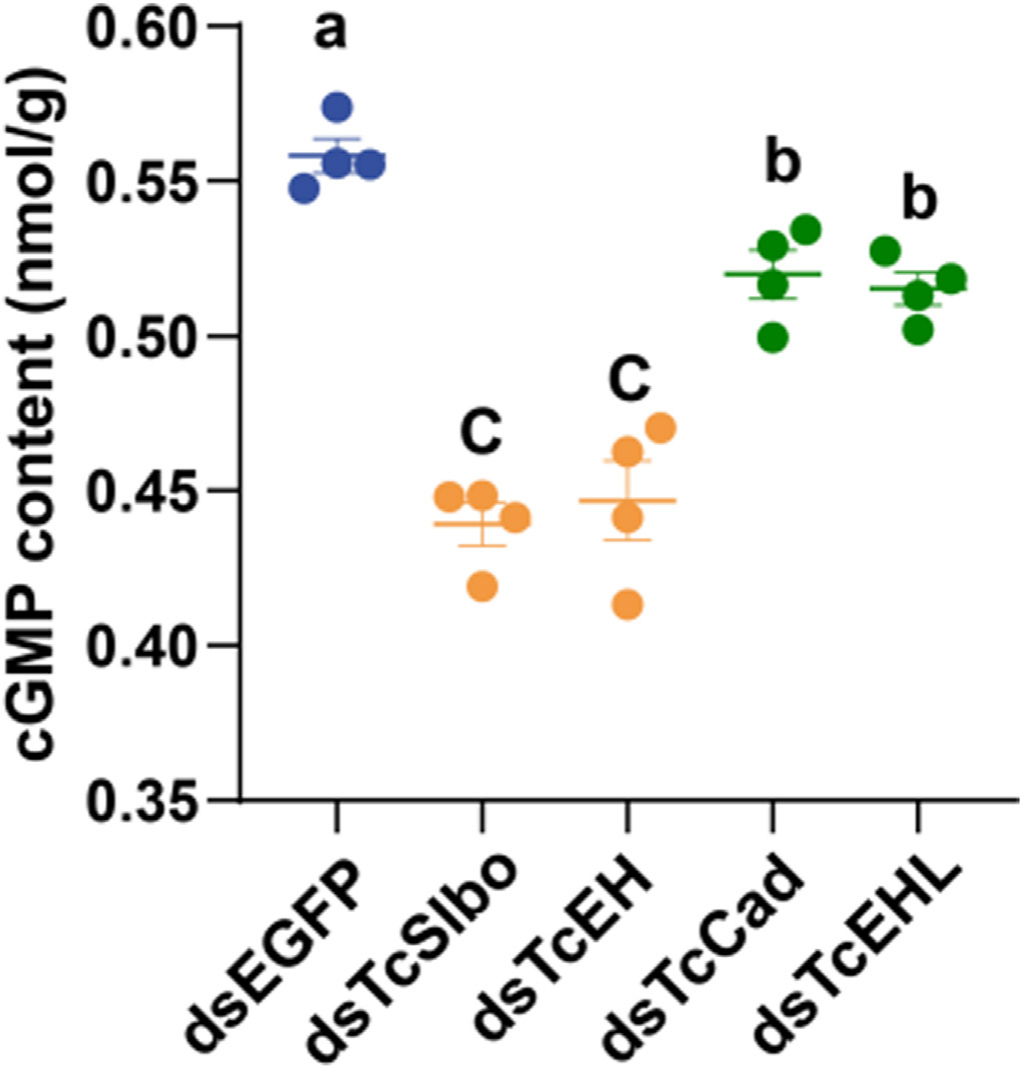
**Effects of *TcSlbo* and *TcCAD* on cGMP levels.** Data were presented as the mean ± SE (one-way ANOVA, Tukey's HSD, *p* < 0.05, n = 4).

As a downstream product of *TcEH* and *TcEHR*, cGMP is an important indicator for evaluating the activation effects of *TcEH* and *TcEHR*. In this study, we measured the cGMP levels in 6-day-old pupae and compared the differences between treatment groups. Compared to the control, the cGMP levels of *TcSlbo* and *TcEH* knockdown pupae were significantly reduced by 21.31% and 19.94%, respectively, and the cGMP levels of *TcCAD* and *TcEHL* knockdown pupae were significantly reduced by 6.87% and 7.68%, respectively (Fig. 4).

### Effects of *TcSlbo* and *TcCAD* on the 20E pathways and 20E titers

**Figure 5:**
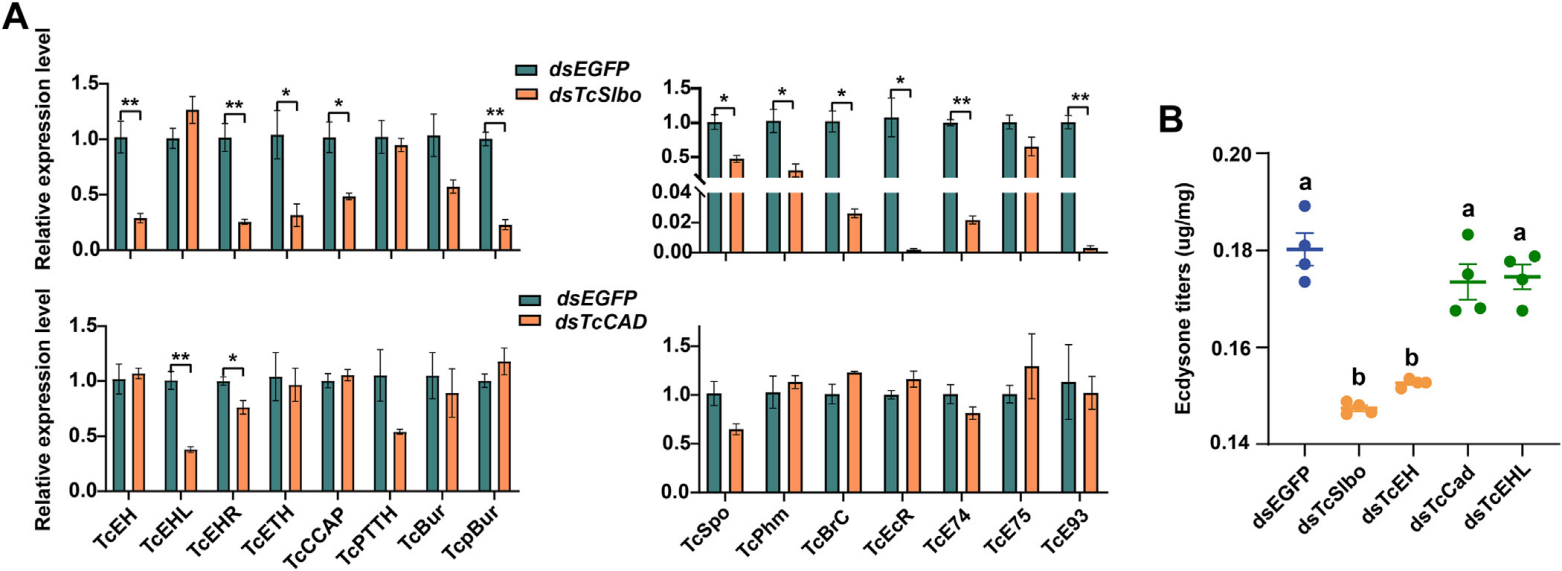
**Effects of *TcSlbo* and *TcCAD* on the 20E pathways and 20E titers.***A*, effects of *TcSlbo* and *TcCAD* on the 20E pathways. Data were presented as the mean ± SE (independent samples T, *p* < 0.05, n = 3). *B*, effects of *TcSlbo* and *TcCAD* on the 20E titers. Data were presented as the mean ± SE (one-way ANOVA, Tukey's HSD, *p* < 0.05, n = 4). 20-E, 20-hydroxyecdysone.

In order to explore the effects of *TcSlbo* and *TcCAD* on the eclosion process of the red flour beetle, we examined the expression of the relevant genes in the 6-day-old pupae. The results showed that the expression of the eclosion pathway related genes *TcEH*, *TcEHR*, *TcETH*, *TcCCAP*, and *TcpBur* were significantly reduced after *TcSlbo* knockdown compared to the double-stranded *EGFP* (*dsEGFP)* control. The expression of the 20E synthesis-related genes *TcSpo* and *TcPhm*, as well as 20E downstream response factors *TcBrC*, *TcEcR*, *TcE74*, and *TcE93*, was also significantly reduced (Fig. 5*A*). Comparatively, the expression of the eclosion pathway–related genes *TcEHL* and *TcEHR* was significantly reduced in *TcCAD* knockdown pupae, but the expression of 20E-related genes did not show any significant changes (Fig. 5*A*).

In addition, we also detected the 20E titers in *TcSlbo* and *TcCAD* knockdown pupae. The results showed that the 20E titers of double-stranded *TcSlbo* (*dsTcSlbo)* and *dsTcEH* were significantly reduced compared to the control, whereas there were no significant changes in the 20E titers of *dsTcCAD* and *dsTcEHL* pupae (Fig. 5*B*).

### Effects of *TcSlbo* and *TcCAD* on the epidermal formation

**Figure 6:**
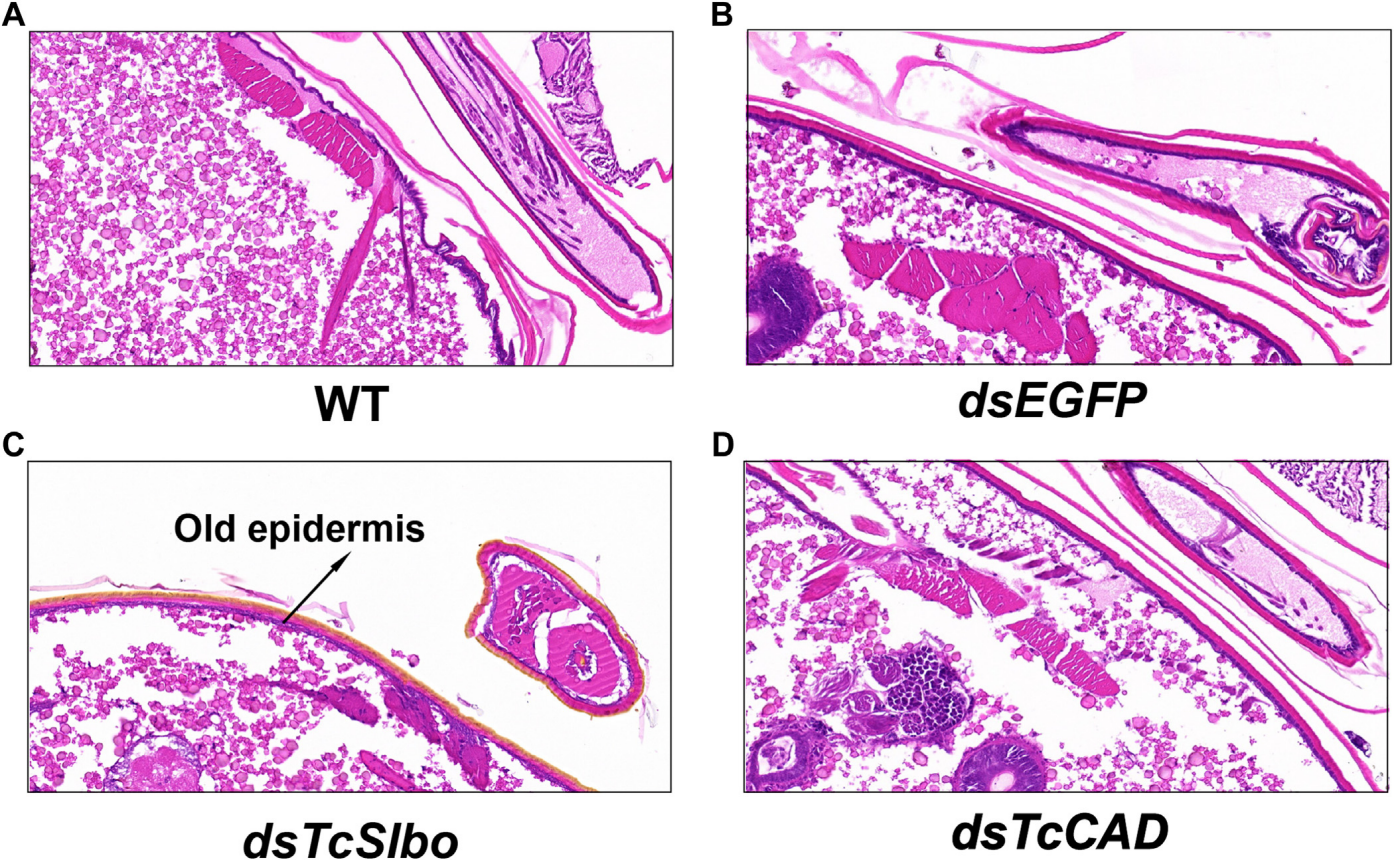
**Effects of *TcSlbo* and *TcCAD* on the epidermal formation in the red flour beetle.***A*, H&E-stained section of the WT late pupal thorax. *B*, H&E-stained section of the late pupal thorax with EGFP knockdown. *C*, H&E-stained section of the late pupal thorax with *TcSlbo* knockdown. *D*, H&E-stained section of the late pupal thorax with *TcCAD* knockdown.

To further investigate the roles of the TFs *TcSlbo* and *TcCAD* in pupal epidermogenesis, we performed paraffin sections and H&E staining of *TcSlbo* and *TcCAD* knockdown pupal thoraxes. The results showed that the old epidermis of *TcSlbo* knockdown pupae was unable to molt normally compared to the control (dsEGFP). However, *TcCAD* knockdown pupae did not show any significant changes compared to the control (Fig. 6).

### *TcSlbo* and *TcCAD* are the response factors for 20E

**Figure 7:**
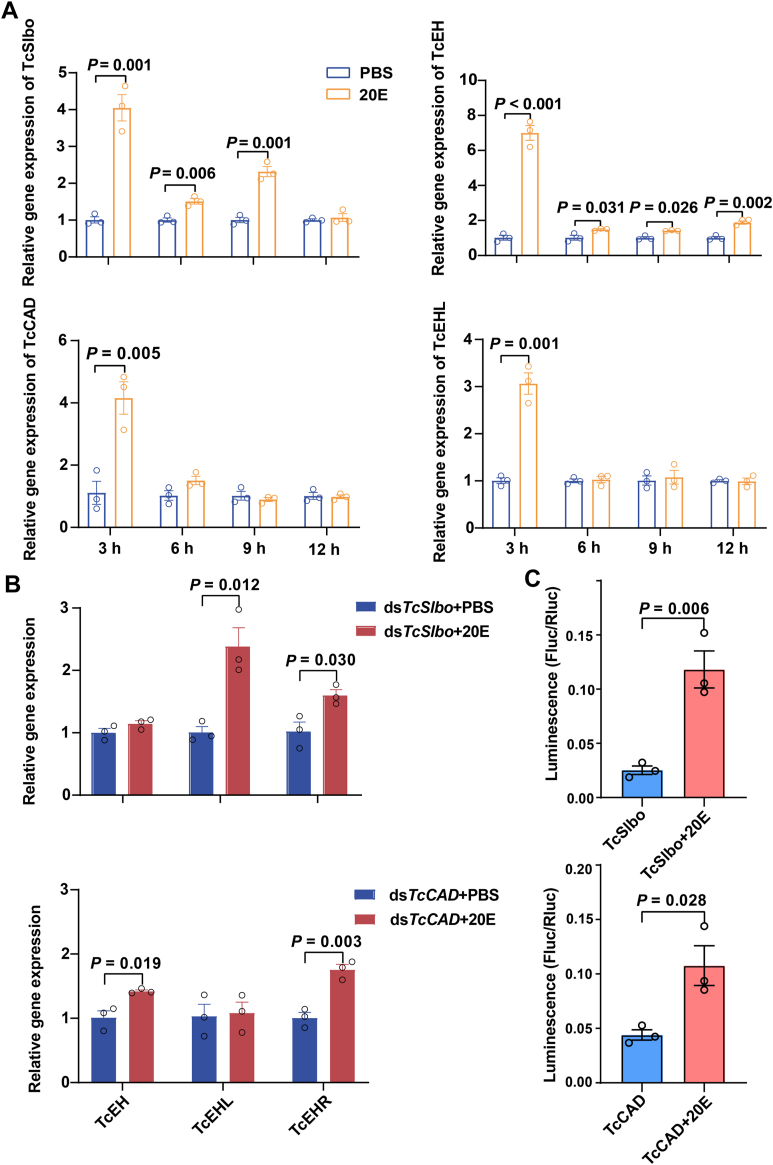
**Effects of 20E on the transcriptional activities of *TcSlbo* and *TcCAD*.***A*, effects of 20E injection for different time periods on the expression of *TcSlbo* and *TcCAD*. *B*, effects of 20E injection on the expression of *TcEH*, *TcEHL*, and *TcEHR* after knockdown of *TcSlbo* or *TcCAD*. *C*, assessment of the transcriptional activity of *TcSlbo* and *TcCAD* in 20E-treated cells using a Dual-Luciferase reporter system. Data were presented as the mean ± SE (independent samples T, *p* < 0.05, n = 3). 20-E, 20-hydroxyecdysone.

In this study, we investigated the effects of the 20E on the activities of the TFs *TcSlbo* and *TcCAD*. The experiment was conducted by injecting 60 ng of 20E into pupae *in vitro* and analyzing the changes in gene expression. Compared to the control group injected with PBS, the expression of the 20E receptor gene *TcEcR* was significantly increased within 12 h, confirming the successful injection of 20E into the pupa (Fig. S1). Notably, the expression of *TcEH* and *TcSlbo* was significantly upregulated at 9 h postinjection, while *TcCAD* and *TcEHL* were significantly increased at 3 h. All genes reached peak expression at 3 h after injection (Fig. 7*A*). We further examined the expression patterns by injecting 300 ng of 20E into *TcSlbo* or *TcCAD* knockdown pupae and analyzed the the expression pattern of *TcEH*, *TcEHL*, and *TcEHR* at 3 h postinjection. In *TcSlbo* knockdown pupae, there was no significant change in the expression of *TcEH* compared to the dsEGFP control group, but the expression of *TcEHL* and *TcEHR* was significantly increased. In *TcCAD* knockdown pupae, there was no significant change in the expression of *TcEHL*, but the expression of *TcEH* and *TcEHR* was significantly increased (Fig. 7*B*). In addition, we also added 1 μM 20E to S2 cells transfected with *TcSlbo* or *TcCAD* plasmid and found that the transcriptional activity of *TcSlbo* and *TcCAD* was significantly increased by 4.69 times and 2.45 times, respectively, compared to cells without 20E (Fig. 7*C*).

## Discussion

Insect molting involves a highly complex biological regulatory network, in which EH plays a central role through the precise regulation of specific TFs (29). In this study, using a Dual-Luciferase reporter system and RNAi technology, we demonstrated for the first time that *TcSlbo* and *TcCAD* are the key TFs that regulate the expression of the *TcEH* and *TcEHL*, and also act as 20E response factors. Our findings indicated that *TcSlbo* not only directly regulates the expression of *TcEH* but also significantly alters the eclosion of *T. castaneum* by modulating cGMP and 20E levels. As the ancestral gene of *TcEH*, *TcEHL* has undergone functional differentiation. Although the knockdown of *TcCAD* did not affect pupal eclosion, it significantly shortened adult lifespan. These results further revealed the critical regulatory roles of *TcSlbo* and *TcCAD* in insect development, providing new insights into the hormonal regulatory networks in insects.

The EH was one of the major hormones regulating larval molting and eclosion in insects (10, 30). Phylogenetic and gene structure analysis suggested that *TcEH* may have arisen from the duplication of the *TcEHL* gene, taking over the role of regulating eclosion, while *TcEHL* gained new features (15). After a long period of evolution, the sequences of *TcEH* and *TcEHL* were significantly different. TFs can bind to specific DNA sequences to regulate transcription. For example, the TFs Antp and POU-M2 increased ecdysone titer by stimulating the transcription of steroidogenic enzyme genes (31). Changes in gene sequences can lead to the recruitment of different TFs (32). In this study, we combined Dual Fluorescein reporter system and RNAi to identify *TcSlbo* and *TcCAD* as the TFs for *TcEH* and *TcEHL*, respectively. These two TFs bind to distinct regulatory elements that initiate the gene expression program of *TcEH* and *TcEHL*, respectively. Previous studies demonstrated that inhibiting *TcEH* expression prevents eclosion in red flour beetles, while inhibiting *TcEHL* expression significantly shortens adult lifespan. Here, similar phenotypic changes were observed when their TFs (*TcSlbo* and *TcCAD*) were knocked down. This finding highlighted the key role of *TcSlbo* and *TcCAD* in the regulation of *TcEH* and *TcEHL* expression (28). In addition, recent research has shown that *SoxC* is also the TF for *EH*, suggesting that *TcSlbo* is not the only TF regulating *TcEH* (21). In some species, the expression of the same gene may be required in response to multiple signals, so there may be several different TFs that bind together to the promoter region of the same gene and coregulate the expression of that gene (33). The 20E affected molting in the *Bombyx mori* by regulating different TFs *POU-M2*, *Antp*, and *Abd-B* in the *CLIP13* promoter region (34). However, we found that the binding site of *TcSoxC* to the promoter region of *TcEH* is located at −1024 bp to −1031 bp not within the *TcEH* high activity promoter fragment.

We found that *TcSlbo* and *TcCAD* were highly expressed in the head and the CNS of 5 to 6 day old pupae, suggesting that they may play a role in neurodevelopment. The expression trends of *TcSlbo* and *TcCAD* were similar to those of *TcEH* and *TcEHL* (28), further suggesting that *TcSlbo* and *TcCAD* are TFs for *TcEH* and *TcEHL*, respectively. Although *TcEH* was regulated by both *TcSoxC* and *TcSlbo*, *TcSlbo* played a crucial role in promoting *TcEH* expression (21). In some studies, the trends of TFs and genes were not consistent. For example, the *FOXO* TF regulated both *Ubc* and *Rotund* genes, and the *FOXO* expression pattern may be affected by a combination of these genes, so the TF was not entirely consistent with the expression pattern of a single target gene (35, 36).

EH is synthesized and released by neurons in the ventromedial region of the brain and, by acting on eclosion hormone receptor (EHR) in the CNS, triggers the production of cGMP, which in turn plays a key regulatory role in neurotransmission, physiological, and developmental processes in insects (37). A significant increase in EH expression caused an increase in cGMP levels during eclosion in *Manduca sexta* (38). In this study, knockdown of the TFs *TcSlbo* and *TcCAD* and their target genes *TcEH* and *TcEHL* in pupa *T. castaneum* pupae resulted in a significant decrease in the cGMP levels, demonstrating that *TcSlbo* and *TcCAD* regulate cGMP levels by binding to the target genes and suggesting that both EH and eclosion hormone-like may act through the EHR receptor. However, different TF knockdown experiments resulted in the *T. castaneum* exhibiting different phenotypes, reflecting the fact that these TFs regulate cGMP production by different mechanisms. Knockdown of *TcSlbo* prevented normal molting of the old epidermis in late pupae, suggesting a strong dependence on cGMP signaling in the preparation for molting. While knockdown of *TcCAD* shortened adult lifespan although it did not affect eclosion, the dependence of these processes on cGMP was relatively low. Such hierarchical and time-dependent regulatory mechanisms have also been demonstrated in *M. sexta* (39), and these findings highlight how organisms can optimize physiological mechanisms to adapt to complex life cycles during evolution.

Studies have shown that EH and 20E work together to ensure that insects molt (40). Using *M. sexta* as an example, 20E induced large accumulation of ETH in Inka cells by binding to the EcR at the onset of successive larval molting behaviors (22). In another study on *L. decemlineata*, *EH* expression was positively correlated with 20E levels and *EH* knockdown-induced molting failure was effectively rescued by external injection of 20E (41). In this study, we found that knockdown of the *TcSlbo* gene triggered a series of cascade reactions in the eclosion signaling pathway in *T. castaneum*. Specifically, the decrease in *TcSlbo* expression reduced the expression of the key target gene *TcEH*. There was a positive feedback mechanism between EH and ETH (42). Consequently, the decrease in *TcEH* expression led to a further decrease in *TcETH* expression, which affected *TcCCAP* and *TcpBur* downstream, ultimately leading to the failure of the pupal eclosion. The absence of any genes in the eclosion pathway may prevent eclosion. For example, *Rhodnius prolixu* larvae lacking *ETH* were unable to completely shed the old epidermis during molting and thus failed to emerge into adults (43). Notably, knockdown of *TcSlbo* also significantly reduced the expression of 20E synthesis genes (*Tcspo* and *Tcphm*) and downstream response factors (*TcBrC*, *TcEcR*, *TcE74*, and *TcE93*), and induced a decrease in 20E titers. As the positive feedback between ETH and EH was interrupted, the decrease in ETH may have directly affected the synthesis of 20E. A similar feedback regulation was found in the *B. mori*. prothoracicotropic hormone stimulates ecdysone secretion from prothoracic gland and injection of 20E into 5-day-old silkworms on day 4 resulted in a surge in prothoracicotropic hormone (33, 44). In contrast, knockdown of *TcCAD* mainly affected the expression of *TcEHL* and *TcEHR* and did not significantly alter 20E synthesis and the expression of related genes. *TcCAD* may be a more specialized regulator in the eclosion regulatory network, unrelated to the 20E synthesis pathway, but rather affecting adult lifespan through precise regulation of cGMP levels.

TFs are typically regulated by a variety of factors, with signaling molecules being the primary regulators (33). External signaling molecules such as hormones, cytokines, and neurotransmitters affect the activity of TFs through signaling pathways (45). 20E is an important insect hormone known to regulate the activity of several TFs (46). 20E regulated the TFs *MHR4* and *βFTZ-F1* to affect molting in the *M. sexta* (47) and affected oocyte maturation in *B. mori* by regulating the TF *BmKr-h1* (48). In this study, the expression of *TcSlbo* and *TcCAD* and their target genes *TcEH* and *TcEHL* was significantly increased at specific times after injection of 20E into WT pupae. 20E regulated molting and metamorphosis by binding to EcR and activating a number of TFs for downstream genes (49). We found that injection of 20E into *TcSlbo* or *TcCAD* knockdown pupae significantly increased the expression of *TcEHL* and *TcEHR* or *TcEH* and *TcEHR*. Furthermore, the addition of 20E to S2 cells overexpressing *TcSlbo* and *TcCAD* significantly enhanced the transcriptional activities of *TcSlbo* and *TcCAD*. Therefore, we considered *TcSlbo* and *TcCAD* to be 20E response genes. Zhou *et al*. (24) also showed that injecting 20E into *E. carinicauda* during the premolt period increased the expression level of *EcEH*, which in turn significantly accelerated the molting process and increased the rate of molting. 20E and EH were both key regulatory hormones in insect molting and metamorphosis, and together they regulated insect development and metamorphosis through precise timing and concentration changes (50).

**Figure 8:**
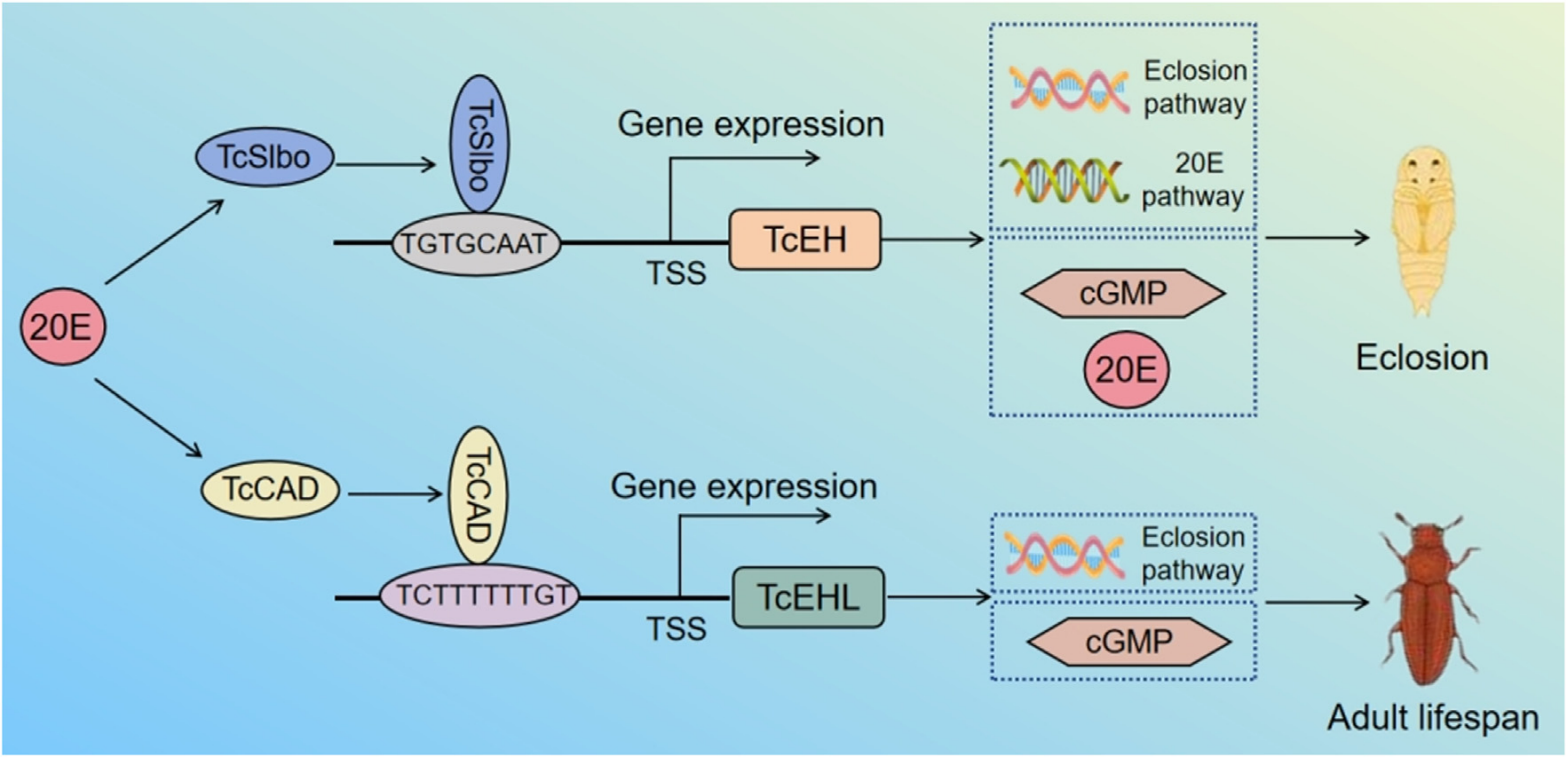
**A model for the transcriptional regulation of the *TcEH* and *TcEHL* genes.** The *TcEH* and *TcEHL* of *Tribolium castaneum* have different transcriptional regulatory mechanisms during eclosion. 20E affects the expression of *TcEH* and *TcEHL* by regulating the transcriptional activity of the transcription factors *TcSlbo* and *TcCAD*, respectively. Decreased expression of both *TcEH* and *TcEHL* reduces the levels of the second messenger cGMP, which affects eclosion and 20E pathway gene expression, ultimately altering insect eclosion and adult lifespan.

In this study, we have demonstrates for the first time that *TcSlbo* and *TcCAD* are the major TFs regulating *TcEH* and *TcEHL*, respectively. *TcSlbo* significantly reduced cGMP and 20E levels by regulating the expression of genes in the eclosion and molting signaling pathway, resulting in a failure to eclosion. Whereas *TcCAD* mainly affected the expression of *TcEHL* and *TcEHR*, slightly reduced cGMP levels and thus shortened adult lifespan. In addition, we have shown that *TcSlbo* and *TcCAD* function as 20E responsive genes. By revealing the complex molecular interactions and functional differentiation in biological evolution, this study not only enhances the understanding of biological functions of EHs but also offered the possibility of developing new pest control strategies (Fig. 8*C*). However, there are challenges in translating these molecular biology tools into agricultural practice. Future research must address these challenges to ensure the long-term effectiveness of these pest control strategies.

## Materials and methods

### Insect strains

The WT strain Georgia-1 of *T. castaneum* was fed on flour containing 5% yeast and reared in an artificial climate chamber. Environmental conditions: temperature, 30 ± 1 °C; relative humidity, 40 ± 5%; photoperiod (light:dark) = 14 h:10 h (51).

### Isolation of total RNA and reverse transcription

Total RNA was extracted from different stages and different tissues of the *T. castaneum* using the TRIzol Reagent kit (CWBIO). RNase-free DNase I (Takara) was used to remove genomic DNA.

The integrity and quality of total RNA were checked using a NanoDrop 2000c spectrophotometer (Thermo Fisher Scientific) and 2% agarose gel electrophoresis. The concentration of the extracted samples was diluted to 1000 ng, and complementary DNA (cDNA) was synthesized using the reverse-transcription system kit (CWBIO).

### Reverse transcription quantitative real-time PCR

The quantitative reverse transcription polymerase chain reaction primers were designed using Oligo7 software (http://www.oligo.net/index.html) (Table S1), and the internal reference gene was ribosomal protein S3 (rps3, GenBank: CB335975) (52). The relative expression levels of the target genes in the samples were detected using the GoTaq quantitative PCR (qPCR) Master Mix Kit (Vazyme). The running program of the fluorescence qPCR instrument (Thermo Fisher Scientific) was as follows: 95 °C for 30 s, 40 cycles of amplification at 95 °C for 10 s and 60 °C for 30 s, 95 °C for 15 s, 60 °C for 1 min, and 95 °C for 15 s rps3 was used as an internal reference gene to normalize the gene expression level using the 2∧^-ΔΔCt^ method (53). Each sample contained three biological replicates and three technical replicates.

### Prediction of TFs for the *TcEH*/*TcEHL* promoter

The *TcEH* and *TcEHL* promoter sequences were obtained from the *T. castaneum* genome (https://ibeetle-base.uni-goettingen.de) and amplified using Phanta Max Super-Fidelity DNA polymerase (Vazyme). *Cis*-regulatory elements and TFs were predicted using the TANSFAC database (http://genexplain.com/transfac/).

### Dual fluorescein reporter experiment

*TcEH* and *TcEHL* promoter fragments of different lengths were amplified from the *T. castaneum* genome and cloned into the pGL3-basic plasmid containing the firefly luciferase gene using the ClonExpress II One Step Cloning Kit (Vazyme). The predicted TF sequences were cloned into the pAc5.1/V5-His b (pAc5.1b) plasmid and sent to Sangon Biotech for sequencing. Plasmids were extracted using MolPure Endo-free Plasmid Mini Kit (YEASEN). The primers used for plasmid construction were listed in Table S1.

Twelve hours before transfection, S2 cells were cultured in 24-well culture plates at 28 °C. Empty pGL3-basic and pGL3-promoter were transfected separately or cotransfected with the pAc5.1b-TFs plasmid into S2 cells, using X-tremeGENE HP DNA transfection reagent (Roche). The internal reference plasmid was phRL-TK. Cells were collected 48 h after plasmid transfection or 24 h after plasmid transfection by replacement with medium containing 1 μM 20E for 24 h. Relative luciferase activity was assayed using the Dual-Luciferase reporter assay kit (Vazyme) and Microplate Reader (BioTek). All dual fluorescein reporter experiments were independently repeated three times.

### dsRNA synthesis and injection

Gene-specific primers were designed with the T7 promoter (Table S2), and PCR amplification was performed using total cDNA. The EGFP fragment was also amplified from the plasmid template containing the gene encoding EGFP using specific primers with the T7 promoter. The amplified products were cut and recovered using a gel extraction kit (Sangon Biotech). The PCR products were used to synthesize dsRNA *in vitro* using the TranscriptAid T7 High Yield Transcription Kit (Vazyme). The quality and integrity of the dsRNA were determined using a Nano Vue spectrophotometer (Thermo Fisher Scientific) and 2% agarose gel electrophoresis. The dsRNA was diluted to 1 μg/μl using DEPC water (Beyotime) containing food coloring (Dinghao), and 200 nl of diluted dsRNA (dsEGFP, dsTcSlbo, dsTcCAD, dsTcEHL, and dsTcEHL) was injected into 1-day-old pupae of the WT strain of *T. castaneum* using a microinjector (WPI). Total RNA was extracted from 5-day-old pupae, reverse-transcribed into cDNA, and the inhibition of gene expression was analyzed by qPCR using dsEGFP-injected *T. castaneum* as a control. The experiment was performed in three independent biological replicates.

### Phenotypic statistics

The number of eclosions and adult lifespan of *T. castaneum* were recorded in the dsEGFP injection control and test groups (knockdown target gene). The eclosion insects were photographed using a stereomicroscope (Leica). Five independent biological replicates were performed with 20 pupae per group.

### cGMP concentration and 20E titer assays

The cGMP concentrations and 20E titers were determined in 5-day-old pupae injected with dsEGFP, dsTcSlbo, dsTcCAD, dsTcEHL, and dsTcEHL. The specific method was as follows: 50 mg of pupae were weighed separately, ground thoroughly in liquid nitrogen, mixed with 450 μl PBS, and centrifuged at 5000 *g* for 10 min. The cGMP concentration and 20E titer in the supernatant were detected according to the methods of the cGMP content kit and the insect ecdysteroid assay kit (ZCIBIO). Each experiment was repeated six times.

### 20E treatment

A total of 0.2 μl of 20E (0.3 μg/μl, Aladdin) was injected into 5-day-old of pupae, and the same volume of PBS was injected as a control using microinjector. After the treatment, the pupae were collected at 3 h, 6 h, 9 h, and 12 h, frozen in liquid nitrogen, and stored at −80 °C for later use. In addition, 0.2 μl of 20E (1.5 μg/μl) was injected into 5-day-old target gene knockdown pupae, and the same volume of PBS was also injected as a control. The Pupae were collected 3 h after injection for follow-up testing. Total RNA was extracted from the samples, reverse transcribed into cDNA and target gene expression was detected by quantitative reverse transcription polymerase chain reaction.

### Staining of H&E paraffin sections

The 1-day-old pupae were injected with dsEGFP, dsTcSlbo, and dsTcCAD. Five days later, after washing three times with 1 × PBS, the insect bodies were punctured with an injection needle to create approximately 20 tiny wounds. The samples were then fixed in 4% paraformaldehyde for 48 h. The fixed samples were sent to Nanjing Youmeng Biotechnology Co. Ltd for paraffin embedding, sectioning, and H&E staining. Paraffin sections were placed under an electron microscope (Wali nova) for epidermal observation and photography.

### Statistical analysis

All data were expressed as mean ± SEM of at least three biological replicates and were statistically analyzed using SPSS (SPSS 19.0 for windows; SPSS Inc; http://www.ibm.com/cn-zh/spss). Data were tested for normal distribution using the Shapiro–Wilk test. If the data conformed to normal distribution, the independent samples *t* test or one-way ANOVA with the Tukey's honestly significant difference test (Tukey HSD) was used, and if the distribution was not normal, the Mann–Whitney *U* test or the Kruskal–Wallis rank sum test was used (54).

## Data availability

All data are contained within the article.

## Supporting information

This article contains supporting information.

## Conflict of interest

The authors declare that they have no conflicts of interest with the contents of this article.
