# Supplementary material for: Novel regulation pathway of eclosion hormones in Tribolium castaneum by distinct transcription factors through the initiation of 20-hydroxyecdysone
Source: J Biol Chem. 2024 Oct 17;300(11):107898. doi: 10.1016/j.jbc.2024.107898 (PMC11650714; doi:10.1016/j.jbc.2024.107898)
Supplement: Supporting information [file mmc1.pdf]

Supporting Information

Novel regulation pathway of eclosion hormones in *Tribolium castaneum* by distinct transcription factors through the initiation of 20-Hydroxyecdysone.

Huiling Zhou[1], Gaoke Lei[2], Yusi Li[1], Peng Chen[1], Zhiping Liu[1], Chengjun Li[1], and Bin Li[1,*]

[1] College of Life Sciences, Nanjing Normal University, Nanjing 210046, China

[2] Institute of Plant Protection, Fujian Academy of Agricultural Sciences, Fuzhou 350013, China

[*]Corresponding author email address: libin@njnu.edu.cn

TABLE OF CONTENTS

Figure S1. Effects of in vitro injection of 20E for different time periods on the *TcEcR* expression. Data were presented as the mean ± SE (Independent samples T, $P < 0.05$, n = 3).

Table S1. Primers for recombinant vector construction.

Table S2. Primers for qRT-PCR and RNAi.

Table S3. Schematic representation of potential transcription factor binding sites in the *TcEH/TcEHL* promoter region.

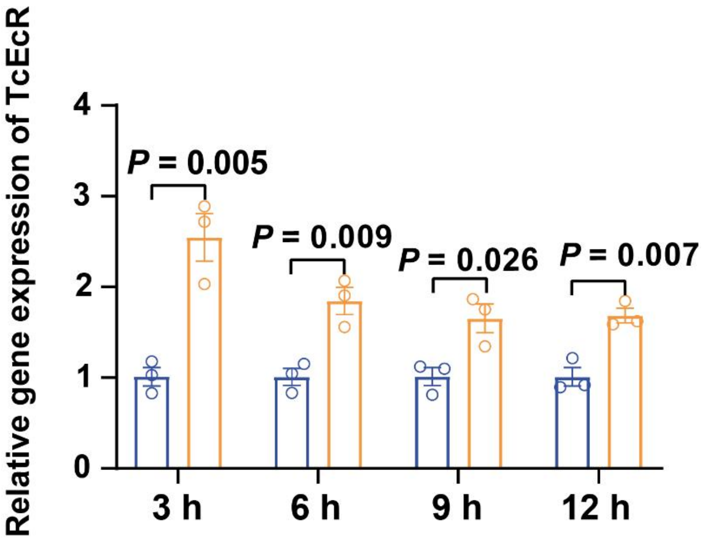

Figure S1. Effects of in vitro injection of 20E for different time periods on the *TcEcR* expression. Data were presented as the mean ± SE (Independent samples T, $P < 0.05$, n = 3).

Table S1. Primers for recombinant vector construction.

Primer Name	Primer sequence 5′-3′
TcEH-pGL3-Fa (-319/+1)	ctctatcgataggtaccCAACATCATCGTCGCGTTTAG
TcEH-pGL3-Fb (-583/+1)	ctctatcgataggtaccCAGTCCATAATCCTGCAACATC
TcEH-pGL3-Fc (-713/+1)	ctctatcgataggtaccCCTGGTTCAGCTAGGCAAC
TcEH-pGL3-Fd (-925/+1)	ctctatcgataggtaccAACCGTAAATATCCAGAAAGCC
TcEH-pGL3-Fe (-1256/+1)	ctctatcgataggtaccCGTCAAACGTATTGCAGAGG
TcEH-pGL3-Fd (-1535/+1)	ctctatcgataggtaccCGTAAACTATAGGCAAAGAAGCAC
TcEH-pGL3-Fd (-1989/+1)	ctctatcgataggtaccGCCACTTGATCTCATCAGTCATAAC
TcEH-pGL3-R	ctctatcgataggtaccCACTGTCCATCTCCACCAAATC
TcEHL-pGL3-Fa (-149/+1)	ctctatcgataggtaccGAGGTGATGTCAGGGGTTAGTATC
TcEHL -pGL3-Fb (-316/+1)	ctctatcgataggtaccTTGGCAATTACTTGTGGGCG
TcEHL -pGL3-Fc (-518/+1)	ctctatcgataggtaccGAGGTGATGTCAGGGGTTAGTATC
TcEHL -pGL3-Fd (-1053/+1)	ctctatcgataggtaccTACGATGCGGGAGTTTAGCG
TcEHL-pGL3-Fe (-1574/+1)	ctctatcgataggtaccGTTGCAAGAGCTATCGTTTCTGC
TcEHL-pGL3-Fd (-2005/+1)	ctctatcgataggtaccTTCTCTTGTTTTATTCACCCCATGC
TcEHL-pGL3-R	ctctatcgataggtaccTGCGGTGGTTGAAATGTAAATGAC
TcDeaf1-pAc-F	tagtccagtgtggtgATGGAGAACTCAAACTCGGAAGG
TcDeaf1-pAc-R	gaagggccctctagaTTATTGTTGCTCTCCGCTTTCAAC
TcPrd-pAc-F	tagtccagtgtggtgATGGATATGAGACCGATCTTTGGC
TcPrd-pAc-R	gaagggccctctagaTTACGCCCAGTAATTCTTGGGC
TcCAD-pAc-F	tagtccagtgtggtgATGGTCTCGTACTACAACTCGACC
TcCAD-pAc-R	gaagggccctctagaTCATATGAGTTGTGAGGGAATTAGGGC
TcOptix-pAc-F	tagtccagtgtggtgATGAACGCCCCAAACCCC
TcOptix-pAc-R	gaagggccctctagaTTACTCGGGATGGCTGTAGAAAATATG
TcSlbo-pAc-F	tagtccagtgtggtgATGGATTCTCCCCAAATGTACGAC
TcSlbo-pAc-R	gaagggccctctagaCTACGGTCCGTATTCGTGCAAC
TcSip1-pAc-F	tagtccagtgtggtgATGTCGGACGACGAGATGG
TcSip1-pAc-R	gaagggccctctagaTTACGCCATGTCTAACAGTCTGTTC
TcBtd-pAc-F	tagtccagtgtggtgATGATGGCTTCCTCGGTCC
TcBtd-pAc-R	gaagggccctctagaTTATTGCTCCCCCAAAGTAATGAACC
TcCG4328-pAc-F	tagtccagtgtggtgATGTTGGAATTTTACCCGAACTTAAACC
TcCG4328-pAc-R	gaagggccctctagaGCATGAGGTAGAGCTTATCAATTGG
TcCaup-pAc-F	tagtccagtgtggtgATGGCAGCCTATGCGCAG
TcCaup-pAc-R	gaagggccctctagaTTAACACGGGCTCCACACC
TcFd59a-pAc-F	tagtccagtgtggtgATGGACGATGGGTGTCGC
TcFd59a-pAc-R	gaagggccctctagaTCACCTCGTCCAGTCGTCG

Table S2. Primers for qRT-PCR and RNAi.

Primer Name	Primer sequence 5′-3′
RNAi	
dsEGFP-F	AAGTTCAGCGTGTCCG
dsEGFP-R	CACCTTGATGCCGTTC
dsTcEH-F	taatacgactcactatagggGAAATTTCCTCGTCCTTTTGCT
dsTcEH-R	taatacgactcactatagggTCAAAAAGGGCGCAATCGAA
dsTcEHL-F	taatacgactcactatagggGCCCACAACCACCTTGTTTTT
dsTcEHL-R	taatacgactcactatagggACACTGCACGCAATTTGTGA
dsTcCAD-F	taatacgactcactatagggCCCAAATGTGGCACCATCAC
dsTcCAD-R	taatacgactcactatagggTGTCTTTCGTCCGGGTCTTG
dsTcOptix-F	taatacgactcactatagggCTCAACGGACGACCTCACAA
dsTcOptix-R	taatacgactcactatagggGTTTGGCCTTGTCCTTGCTG
dsTcSlbo-F	taatacgactcactatagggCTCCAGCAGTACCAGCAACA
dsTcSlbo-R	taatacgactcactatagggTGCTGGTTGAGCATCATCGT
dsTcBtd-F	taatacgactcactatagggAGGATGTACGACCCCTACCC
dsTcBtd-R	taatacgactcactatagggAGATGCGAGGTTTTGCCGTA
dsTcCaup-F	taatacgactcactatagggGCCCTCCAAGCCGAAGATTT
dsTcCaup-R	taatacgactcactatagggATGTCGCAGCCTTTTAGTGC
qRT-PCR	
qrps3-F	TCAAATTGATCGGAGGTTTG
qrps3-R	GTCCCACGGCAACATAATCT
qTcEH-F	AGACGCAACGCATTAGGACA
qTcEH-R	ACTGGACTAAGTCGGGCGAA
qTcEHL-F	CAATCCCAGTTTGCATCACAAAT
qTcEHL-R	GCCCAAAGTTCCTGCATTGTT
qTcEHR-F	TGTGTCGGCATATGTGGGG
qTcEHR-R	ACACCCAGGACGAAATACTCG
qTcCAD-F	CAAGACCCGGACGAAAGACA
qTcCAD-R	TCCGCCGGATTGTGATGTAG
qTcOptix-F	ATGGAAGTCGAACCACCGAC
qTcOptix-R	GTACTCGGCTAAGGGGATGC
qTcSlbo-F	ACAATCATCAGCAGGGCCAA
qTcSlbo-R	GTCGTCTGCTTCGAAATGCC
qTcBtd-F	TTGAGGACACATACGGGGGA
qTcBtd-R	TTCCCGCACACATTCTCACT
qTcCaup-F	CAAGTTTGCCCACAACCGAA
qTcCaup-R	CGAACCCTCCGTTCATGCTT
qTcPTTH-F	ATGACGAAGTGGATGATCGGTG
qTcPTTH-R	GGCCGTGAAGGATGGTAATAAGG
qTcETH-F	ATGCGTCGTTACCAAATTCTC
qTcETH-R	CTACATCACGTAATAGAATTTCGG
qTcBur-F	ACCGACGAGTGCCAGGTC

qTcBur-R	ATTATTGAGAGTGAGACTTGAG
qTcpBur-F	ATCAAAGAGGAATTCGACGAAT
qTcpBur-R	TTATCGGCTGAAATCGCCAC
qTcCCAP-F	ATGACCACGGCCAAACTCTTC
qTcCCAP-R	TTAAATGTAGCAGGGCGGGAG
qTcSpo-F	GGGACGAGCCTGGACTGTT
qTcSpo-R	CCCGTGCTGAAAGGAATGA
qTcPhm-F	AAGAATGTGTGTCGGTGATGAA
qTcPhm-R	TCGTGAGGTTTCGGAGTTAGTG
qTcBrC-F	CACAACACTTCTGTCTGCGGTG
qTcBrC-R	CACAGGGTGTTTGCAAGGAG
qTcEcR-F	GATGGATGGCGAAGATCAGT
qTcEcR-R	ACTTCGCTGGAACATGCTTT
qTcE74-F	CTACCTTCTCCAGCAGCACCAC
qTcE74-R	AGTGGCCGCCTGGATCA
qTcE75-F	GCCACCATCGTGTTCAAGTC
qTcE75-R	GGAAGAAGCCCTTGCATCCT
qTcE93-F	CTCTCGAAAACTCGGTTCTAAACA
qTcE93-R	TTTGGGTTTGGGTGCTGCCGAATT

Table S3. Schematic representation of potential transcription factor binding sites in the *TcEH/TcEHL* promoter region.

Gene	Binding motif	Transcription factor	Sequence position (rel. to TSS)
TcEH	CCGTGGCCGAAG	TcPrd	-585/-596
	GCCGAA	TcDeaf1	-586/-591
	ACATAAACAA	TcCAD	-662/-671
	CACATAAACAA	TcSip1	-662/-672
	TGATA	TcOptix	-671/-675; -674/-678
	TGTGCAAT	TcSlbo	-683/-690
TcEHL	CAATAAA	TcCG4328	-231/-237
	CAAAAATAACAAT	Tcfd59A	-234/-246
	TAACA	TcCaup	-236/-240
	TCTTTTTTGT	TcCAD	-286/-295
	TTGTGGGCGTT	TcBtd	-295/-305
